# Experimental Testing, Manufacturing and Numerical Modelling of Composite and Sandwich Structures (Second Edition)

**DOI:** 10.3390/ma18184372

**Published:** 2025-09-19

**Authors:** Raul D. S. G. Campilho

**Affiliations:** 1 CIDEM, ISEP—School of Engineering, Polytechnic of Porto, R. Dr. António Bernardino de Almeida, 431, 4200-072 Porto, Portugal; raulcampilho@gmail.com; 2 INEGI—Institute of Science and Innovation in Mechanical and Industrial Engineering, Pólo FEUP, Rua Dr. Roberto Frias, 400, 4200-465 Porto, Portugal

Composite and sandwich structures are nowadays indispensable in engineering applications where lightweight, durability, and multifunctionality are critical [1]. Due to these requirements, aerospace, automotive, marine, energy, construction, and biomedical industries increasingly rely on these materials due to their superior strength-to-weight ratios, corrosion resistance, and geometric freedom [2,3]. The versatility of these materials arises from the combination of different reinforcements (fibers, nanoparticles, metallic foils) with diverse matrices, thus enabling customized properties for specific applications [4,5]. Despite this progress, several challenges in the design of composite and sandwich structures persist. The high production costs, complex repairability, and difficulties in accurately predicting failure constitute barriers to the wider application of these solutions [6,7]. Moreover, sandwich structures, while offering excellent stiffness and energy absorption, are still vulnerable to impact, delamination, and durability issues under environmental exposure [8]. Recent research has therefore focused on advanced manufacturing techniques (3D printing, digital manufacturing, friction stir processing) [9,10,11], bio-based and recyclable composites [12], multifunctional systems with embedded sensing or actuation, and robust numerical models capable of simulating damage initiation and propagation with high accuracy [13,14,15]. This Special Issue, entitled “Experimental Testing, Manufacturing and Numerical Modelling of Composite and Sandwich Structures (Second Edition)”, brings together 19 papers that explore these aspects from experimental, manufacturing, and numerical perspectives. The contributions are divided into five topics, described below, along with selected papers highlighting recent advances and future directions in this field.

**Advanced Manufacturing and Processing of Composites**

The development of advanced processing methods aims to make composites more accessible, sustainable, and customizable. Traditional techniques such as autoclave curing, resin transfer molding, and filament winding, while capable of producing high-quality components, are often limited by high costs, energy demands, and long cycle times [16]. Moreover, mass production remains a barrier for industries such as automotive and renewable energy, where cost competitiveness is critical [17]. On the other hand, emerging methods such as additive manufacturing (AM), friction stir processing (FSP), ultrasonic-assisted casting, and hybrid fabrication are being explored to surpass these limitations [18,19]. These processes enable complex geometries and graded architectures and enable embedding sensors or reinforcements during fabrication. At the same time, computational tools and artificial intelligence are increasingly used to optimize process parameters, microstructures, and resulting properties [20]. Sarmah and Gupta [21] present a systematic review of recent fabrication advancements in metal matrix composites (MMCs), by covering both traditional and advanced methods such as friction stir processing, ultrasonic-assisted casting, and additive manufacturing. Their work also discusses artificial intelligence applications in manufacturing optimization. Quinlan et al. [22] developed nanocrystalline cobalt–iron-substituted gadolinium-doped ceria (CoxFe1−xGDC) powders as a novel solid oxide fuel cell (SOFC) anode, using a co-precipitation route with ammonium tartrate. The study combines extensive characterization techniques and demonstrates promising electrochemical performance.

**Mechanical Behavior, Damage and Impact Resistance**

The application of composites in safety-critical industries such as aerospace and automotive highly depends on their mechanical performance and damage tolerance [23]. Failure in composites is far more complex than in isotropic materials, involving multiple interacting mechanisms: matrix cracking, fiber breakage, delamination, fiber–matrix debonding, and interlaminar shear [24]. The anisotropy and heterogeneity of composites add to the difficulty in predicting failure. Equally, sandwich structures often fail through skin–core debonding, core shear, or local buckling, which can initiate catastrophic collapse [25]. A particularly important issue is impact resistance, as both low-velocity (tool drops, runway debris) and high-velocity impacts (bird strikes, ballistic hits) may introduce hidden internal damage that drastically reduces the residual strength [26]. Research is therefore focused on hybridization strategies (e.g., fiber–metal laminates, cork cores, rubber interlayers), bio-based solutions, and advanced constitutive models to capture post-impact behavior [27]. Ursache et al. [28] studied carbon–aramid composites with and without rubber cores under low-velocity impact and water absorption. The results show how moisture severely reduces absorbed energy, especially for rubber-core systems. Zhang et al. [29] investigate hybrid fiber–metal laminates combining thermoplastic CFRP with aluminum alloy, subjected to high-velocity impacts. Both experiments and simulations confirm their superior energy absorption compared to conventional laminates.

**Numerical Modelling and Simulation Advances**

Numerical modelling of composites is traditionally limited by the difficulty of simultaneously capturing their heterogeneity, anisotropy, and multi-scale behavior [30]. Conventional FEM has been very successful but is limited due to mesh-dependence in crack propagation, difficulties with multi-scale homogenization, and high computational costs [31,32]. Recent efforts are directed toward meshless methods, multi-scale modelling, digital twins, and AI-enhanced simulations, which aim to balance accuracy and computational feasibility [33,34,35,36]. Modelling matrix cracking, delamination, and fiber breakage requires cohesive elements, continuum damage mechanics (CDM), or phase-field methods, each with its own limitations [37,38,39]. Equally, numerical methods are being linked to structural health monitoring by simulating acoustic emissions, Lamb waves, and vibrational signatures [40]. Rijal et al. [41] simulate acoustic emissions from composite failure modes (matrix cracking, delamination, fiber breakage) via finite element modelling of Lamb waves. Their work includes both experiments and modelling, enhancing structural health monitoring techniques. Cho [42] develops a natural element method (NEM) approach for coupled thermal and mechanical buckling in graphene platelet-reinforced composites. The study highlights the significance of thermo-mechanical interactions in design.

**Sandwich Structures and Structural Dynamics**

Sandwich structures are widely used due to their excellent stiffness-to-weight ratios and energy absorption capabilities [43]. Their mechanical behavior, however, is highly dependent on the interaction between skins, core, and adhesive interfaces [44,45]. Issues such as core shear failure, skin wrinkling, and debonding remain dominant failure modes [46,47]. With the trend toward functionally graded cores, 3D-printed honeycombs, and bio-inspired cellular structures, researchers are also investigating vibration behavior, damping, and adaptive functionalities [48]. Dynamic loading scenarios, such as vibration, acoustic excitation, and impact, are particularly critical in aerospace and automotive settings [49]. Structural health monitoring and active vibration control are therefore gaining importance by researching smart actuators and piezoelectric layers integrated into sandwich beams [50,51]. Raza et al. [52] present a combined numerical and experimental study of vibration control in thin-walled additively manufactured PLA and PLA-composite beams. Macro fiber composites are shown to effectively reduce vibration amplitudes. Bunoiu et al. [53] create magnetorheological suspensions based on lard, gelatin, and carbonyl iron particles, demonstrating tunable dielectric and rheological properties under magnetic fields. This approach points to adaptive structural materials with potential sandwich applications.

**Sustainability, Multifunctionality, and Novel Applications**

Designers of composite structures are increasingly adopting sustainability and multifunctionality as major design principles [54]. Scrapping composites is unsustainable due to the difficulty of recycling thermosets, the energy intensity of fiber production, and the need for petrochemical raw materials [55]. Solutions include using bio-based fibers and resins, recycling aerospace waste prepregs, and extending service life through repair and strengthening [56,57]. Parallel to sustainability, multifunctionality is being pursued by integrating composites with sensing, energy harvesting, or catalytic functions, which enables structures to contribute to functions other than load-bearing [58]. Chanchí-Golondrino et al. [59] apply computational methods to detect asbestos cement in hyperspectral images, proposing efficient algorithms (spectral differential similarity, SDS; Fourier phase similarity, FPS) that combine accuracy with acceptable computational cost. Zhou et al. [60] report the synthesis of Ce-doped α-MnO_2_ catalysts via a hydrothermal method, where Ce incorporation refined the microstructure and enhanced defect formation. Detailed characterization confirmed strong Mn–Ce interactions and electron transfer, favoring Mn^3+^ species.

The contributions in this Special Issue highlight the vitality of composite and sandwich structure research and its interdisciplinary nature. They range from innovations in advanced manufacturing and novel hybrid material systems to new modelling strategies and sustainability-oriented solutions, showing the rapid evolution of the field and its growing relevance across diverse engineering domains. Looking ahead, several promising research topics have been identified:

- Artificial intelligence and machine learning are expected to accelerate the discovery of new composite formulations and predict damage and failure at different scales. Coupled with large experimental datasets, these tools will transform design and reduce development time [61].
- Circular economy strategies will gain importance, especially in aerospace, automotive, and transport, where the reuse of high-grade fibers, recycling of thermoplastic matrices, and upcycling of aerospace prepregs could drastically reduce environmental impact [62].
- The development of multifunctional and smart composites, capable of self-sensing, self-healing, or energy harvesting, will expand the functionality of materials, enable real-time health monitoring, and extend service life [63].
- The integration of digital twins and Industry 4.0 technologies will facilitate real-time monitoring and adaptive performance optimization to ensure safety, efficiency, and predictive maintenance [64].
- Hybrid material systems that combine composites with metals, ceramics, or nano-functional fillers will open up design possibilities for extreme-condition applications, particularly in aerospace, marine, defense, and renewable energy, where materials must withstand complex loading and harsh environments [65].
- Bio-inspired and bio-based composites are expected to grow in relevance, both for sustainability and for their ability to mimic the hierarchical structures and toughening mechanisms found in natural materials such as bone, seashells, or plant fibers [66].

These directions point towards a future where composites and sandwich structures will be lighter and stronger, and also smarter, greener, and more adaptive. As Guest Editor, I would like to express my sincere gratitude to all the authors for their high-quality contributions, which have greatly enriched this Special Issue. I hope that this Special Issue serves as a valuable reference for both researchers and engineers, by providing an insight into the latest advances in experimental testing, manufacturing, and numerical modelling of composites and sandwich structures.
